# SARS‐CoV‐2 variants preferentially emerge at intrinsically disordered protein sites helping immune evasion

**DOI:** 10.1111/febs.16379

**Published:** 2022-02-15

**Authors:** Federica Quaglia, Edoardo Salladini, Marco Carraro, Giovanni Minervini, Silvio C.E. Tosatto, Philippe Le Mercier

**Affiliations:** ^1^ Institute of Biomembranes, Bioenergetics and Molecular Biotechnologies National Research Council (CNR‐IBIOM) Bari Italy; ^2^ Department of Biomedical Sciences University of Padova Italy; ^3^ Swiss‐Prot group SIB Swiss Institute of Bioinformatics Geneva Switzerland

**Keywords:** biocuration, DisProt, IDPs, immune escape, intrinsically disordered proteins, mutations, SARS‐CoV‐2, variants, ViralZone

## Abstract

The SARS‐CoV‐2 pandemic is maintained by the emergence of successive variants, highlighting the flexibility of the protein sequences of the virus. We show that experimentally determined intrinsically disordered regions (IDRs) are abundant in the SARS‐CoV‐2 viral proteins, making up to 28% of disorder content for the S1 subunit of spike and up to 51% for the nucleoprotein, with the vast majority of mutations occurring in the 13 major variants mapped to these IDRs. Strikingly, antigenic sites are enriched in IDRs, in the receptor‐binding domain (RBD) and in the N‐terminal domain (NTD), suggesting a key role of structural flexibility in the antigenicity of the SARS‐CoV‐2 protein surface. Mutations occurring in the S1 subunit and nucleoprotein (N) IDRs are critical for immune evasion and antibody escape, suggesting potential additional implications for vaccines and monoclonal therapeutic strategies. Overall, this suggests the presence of variable regions on S1 and N protein surfaces, which confer sequence and antigenic flexibility to the virus without altering its protein functions.

## Graphical abstract

Intrinsically disordered regions (IDRs) are abundant in the SARS‐CoV‐2 spike glycoprotein S1 subunit and nucleoprotein. Mutations occurring in the 13 main variants, including Omicron, correlate with these IDRs and play a role in immune evasion. Moreover, IDRs in the major antigenic site of the spike glycoprotein suggest a role of intrinsic disorder in escaping the host immune response.

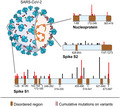

**Abbreviations**
IDP: intrinsically disordered protein
IDR: intrinsically disordered region
N: nucleoprotein
NTD: N‐terminal domain
RBD: receptor‐binding domain
RBM: receptor‐binding motif
S: spike glycoprotein
SARS‐CoV‐2: severe acute respiratory syndrome coronavirus 2
VOC: variant of concern
VOI: variant of interest

## Introduction

Intrinsically Disordered Proteins (IDPs) are a widespread class of diverse proteins characterized by lack of a fixed 3D structure [1]. IDPs are well known players of multiple biological processes, such as nucleic acid binding, signalling, cell cycle regulation, and play a central role in a large number of physiological and pathological processes [2]. Although widely distributed in eukaryotes, the widest content is found among viruses [3], where IDPs have evolved to support virus‐related biological functions [4, 5]. Disordered proteins represent an important class of antigens in a variety of human pathogens and can be targets of protective antibody responses [6].

The presence of protein intrinsic disorder was also highlighted in the Severe acute respiratory syndrome coronavirus 2 (SARS‐CoV‐2) proteome [7, 8, 9]. In particular, both spike glycoprotein (S) and nucleoprotein (N) are nowadays well known to contain functionally relevant disordered regions (IDRs) [7, 8, 9]. Since the onset of the COVID‐19 pandemic, several SARS‐CoV‐2 variants have been identified worldwide [10], affecting the epidemiology of the virus, and playing an important role in pandemic surveillance and control [11, 12]. Mutations that affect the viral genome and potentially impact disease transmission and severity are referred to as variants of concern (VOC) and variants of interest (VOI), and the scientific community is increasingly dedicated to monitoring the emergence of new viral lineages worldwide. The most variable proteins are spike and nucleoprotein, which are also the major antigenic proteins [13].

In this work, we use manually curated structural data to describe the disordered regions of SARS‐CoV‐2—as a collaboration between leading data resources, UniProt [14], ViralZone [15] and DisProt [16, 17]—focusing on the spike protein and nucleoprotein. Many different SARS‐CoV‐2 variants have been observed: there are 1737 lineages described in PANGO (https://cov‐lineages.org/index.html/cite) as of December 2021. We chose to analyse the 13 Variants Of Concern (VOC) and the Variants Of Interest (VOI)—including Omicron—as they represent the most widespread and best adapted to humans (https://www.who.int/en/activities/tracking‐SARS‐CoV‐2‐variants/). We analyse mutation localization for these 13 major variants of the SARS‐CoV‐2 virus and uncover hotspots that correlate not only with disordered regions but also with immune evasion. Finally, we highlight the role of flexible regions in the major antigenic site of the spike protein, suggesting a role of intrinsic disorder in escaping the host immune response.

## Results

### SARS‐CoV‐2 spike and nucleoprotein are enriched in IDRs

Intrinsically disordered proteins are characterized by the presence of unstructured segments, that is, intrinsically disordered regions (IDRs), that lack a stable tertiary structure. Intrinsic disorder in proteins can be identified by several experimental techniques, including biophysical and biochemical methods, the most widely used being X‐ray crystallography, nuclear magnetic resonance (NMR), circular dichroism and small‐angle X‐ray scattering [18, 19]. Using the information available in DisProt, the major repository of manually curated data of IDPs and IDRs from literature data, we investigated the presence of IDRs in the SARS‐CoV‐2 proteins, along with their interactions and functions [16, 17]. By analysing published structures and raw experimental data, we investigated IDR regions in nucleoprotein, spike, E protein, ORF1ab, ORF3a and ORF7a proteins. We focused our analysis on those proteins playing a crucial role in the virus–host interaction, and targets of vaccines and antibodies development, that is, proteins spike and nucleoprotein [20, 21].

Analysis revealed that several regions are omitted in the structures of SARS‐CoV‐2 spike glycoprotein (protein S, DisProt: DP02772) due to their flexibility. No apparent density can be detected for region 455‐490 [7]: this region of the Receptor‐Binding Motif (RBM) is indeed unstructured and flexible in the unbound conformation [7, 8] and undergoes folding‐upon‐binding in the ACE2‐bound form [22, 23].

The IDR between S1 and S2 (673–686) [7] is required for the proteolytic processing essential for the viral entry into host cells [24]. An insertion at position 680–687, that includes the specific furin‐like cleavage motif RRxR, has been shown to be absent in other beta coronaviruses such as SARS‐CoV [25].

Several sterically accessible complex‐type glycans were identified inside the IDRs of SARS‐CoV‐2 spike glycoprotein (N74, N149 and three positions in the unstructured C terminus, N1158, N1173, N1194) as characterized by mass spectrometry experiments [26]. As protein glycosylation is a well‐established strategy adopted by viruses to evade host immunity [27], molecular dynamic simulations highlighted that glycans extensively shield the spike protein surface from antibody recognition [28]. Nevertheless, we found no significant correlation between glycan sites and IDR in spike protein.

SARS‐CoV‐2 nucleoprotein (protein N, DisProt: DP03212) is a 419‐residue multidomain protein characterized by 52% of disorder content that include the unstructured N‐ and C‐termini, along with a disordered flexible linker connecting the RNA‐binding domain (RBD) and the dimerization domain [29]. The disordered N terminus plays a role in liquid–liquid phase separation of protein N, indeed its deletion strongly decreases phase separation in the presence of RNA, while slightly increasing turbidity and droplet formation in the absence of RNA [30]. Similarly, a deletion of the flexible linker (region 174‐247) strongly reduces LLPS‐associated droplet formation and turbidity [30]. NMR titration experiments characterizing the interaction of polyU with the protein N SR‐peptide, region 182‐197 inside the flexible linker that connects the two globular domains, indicate that the interaction strength decreases in the phosphorylated form. Moreover, phosphorylation of full‐length nucleoprotein affects its RNA‐induced phase separation, resulting in a weaker interaction of protein N with RNA and an increased diffusion of the phosphorylated species inside polyU‐induced droplets [31]. The C‐terminal IDR, instead, is not required for nucleoprotein condensation with RNA via LLPS [31]. The N‐terminal and C‐terminal IDRs were also found to be involved in the binding of nucleocapsid‐targeting single‐domain antibodies (sdAbs), sdAbs‐N5 and sdAb‐N6, whose interaction with the nucleoprotein requires the presence of its intrinsically disordered termini [32]. Size‐exclusion chromatography studies of the nucleoprotein in RNA‐bound states and RNA‐free state showed that truncations of its N‐terminal IDR impair the RNA binding and that both the N‐terminal and C‐terminal IDRs contribute to RNA‐binding activity of the SARS‐CoV‐2 nucleoprotein [33]. Finally, the C‐terminal disordered region seems to play a role in droplet formation [33].

### S1 and N mutation hotspots cluster in unstructured regions

**Fig. 1:**
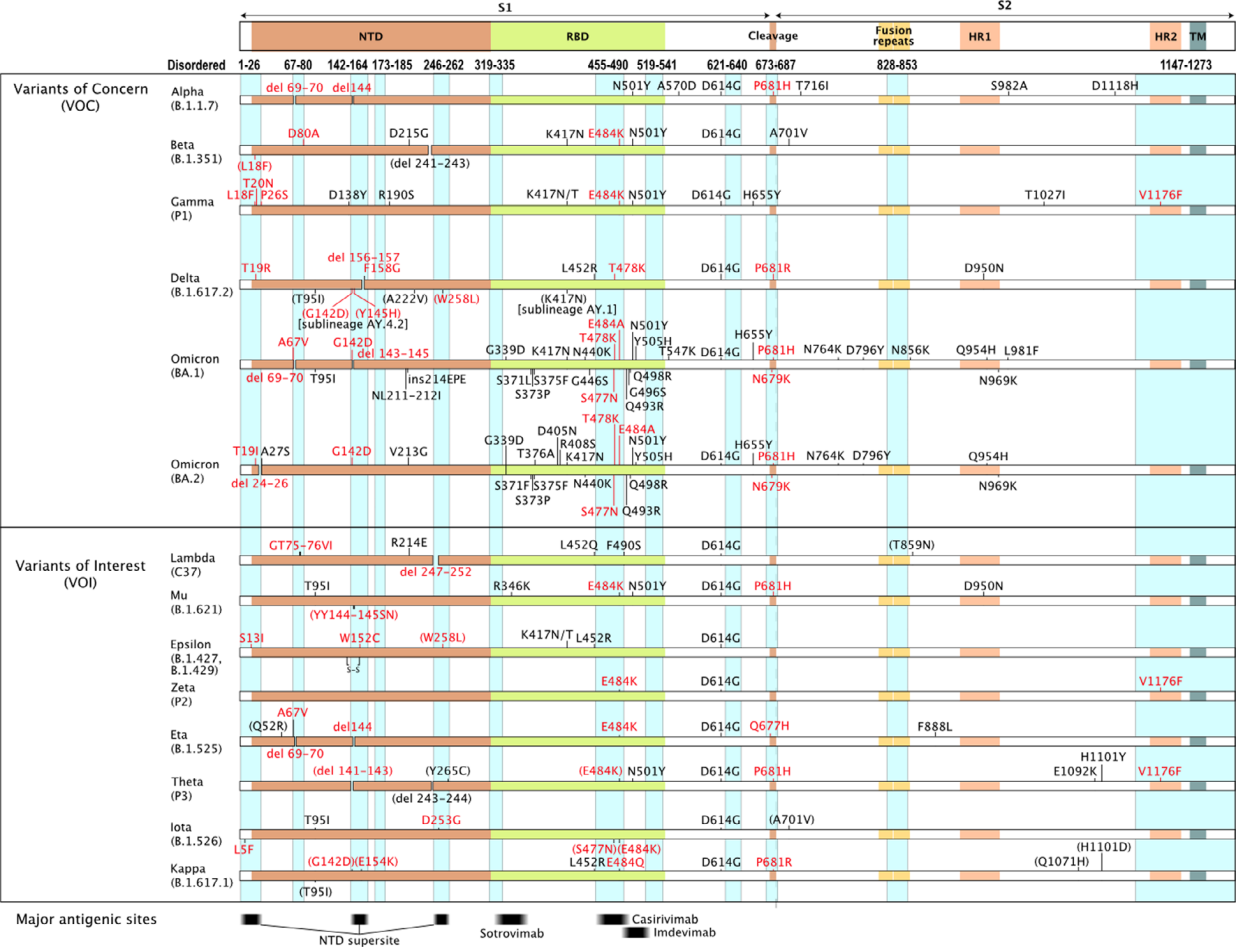
Amino acid changes in the spike protein of Variants of Concern (VOC) Alpha, Beta, Gamma, Delta, Omicron BA.1 and BA.2; Variants of Interest (VOI) Lambda, Mu, Epsilon, Zeta, Eta, Theta, Iota and Kappa. Disordered regions are indicated by cyan columns, and variants in disordered regions are coloured in red. Parentheses indicate variants whose prevalence is < 80% but > 10%. The main regions are annotated: S1 with N‐terminal domain (NTD) and receptor‐binding domain (RBD); S2 with fusion peptides, heptad repeat 1 (HR1) and 2 (HR2) and the transmembrane domain (TM) [73]. Major antigenic sites are shown below with the NTD supersite [56], and monoclonal antibody‐binding regions for sotrovimab [74], casirivimab and imdevimab [75, 76].

**Fig. 2:**
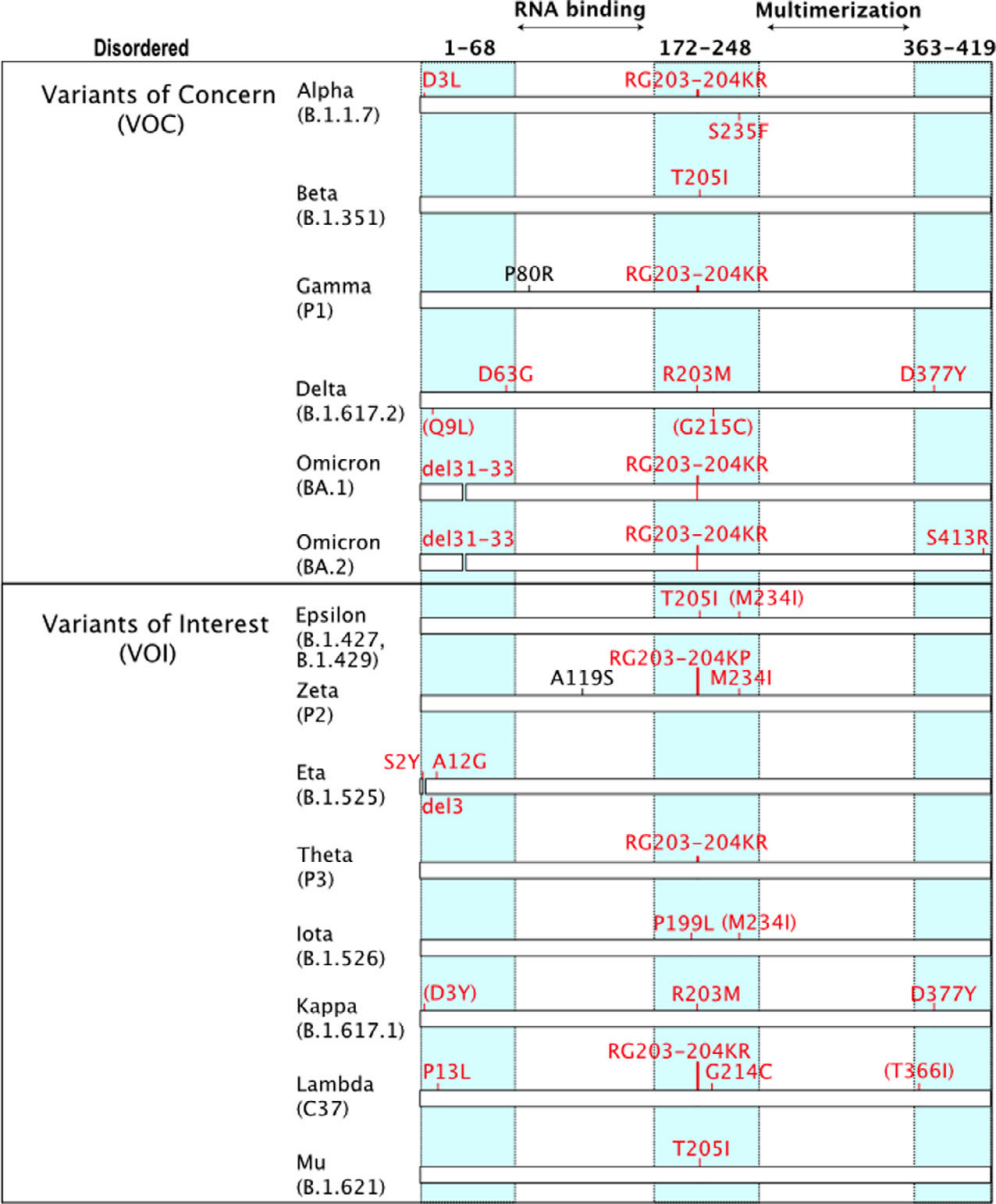
Amino acid changes in the nucleoprotein of Variants of Concern Alpha, Beta, Gamma, Delta, Omicron BA.1 and BA.2; Variants of Interest (VOI) Lambda, Mu, Epsilon, Zeta, Eta, Theta, Iota and Kappa. Disordered regions are indicated by cyan columns, and variants in disordered regions are coloured in red. Parentheses indicate variants whose prevalence is < 80% but > 10%.

**Fig. 3:**
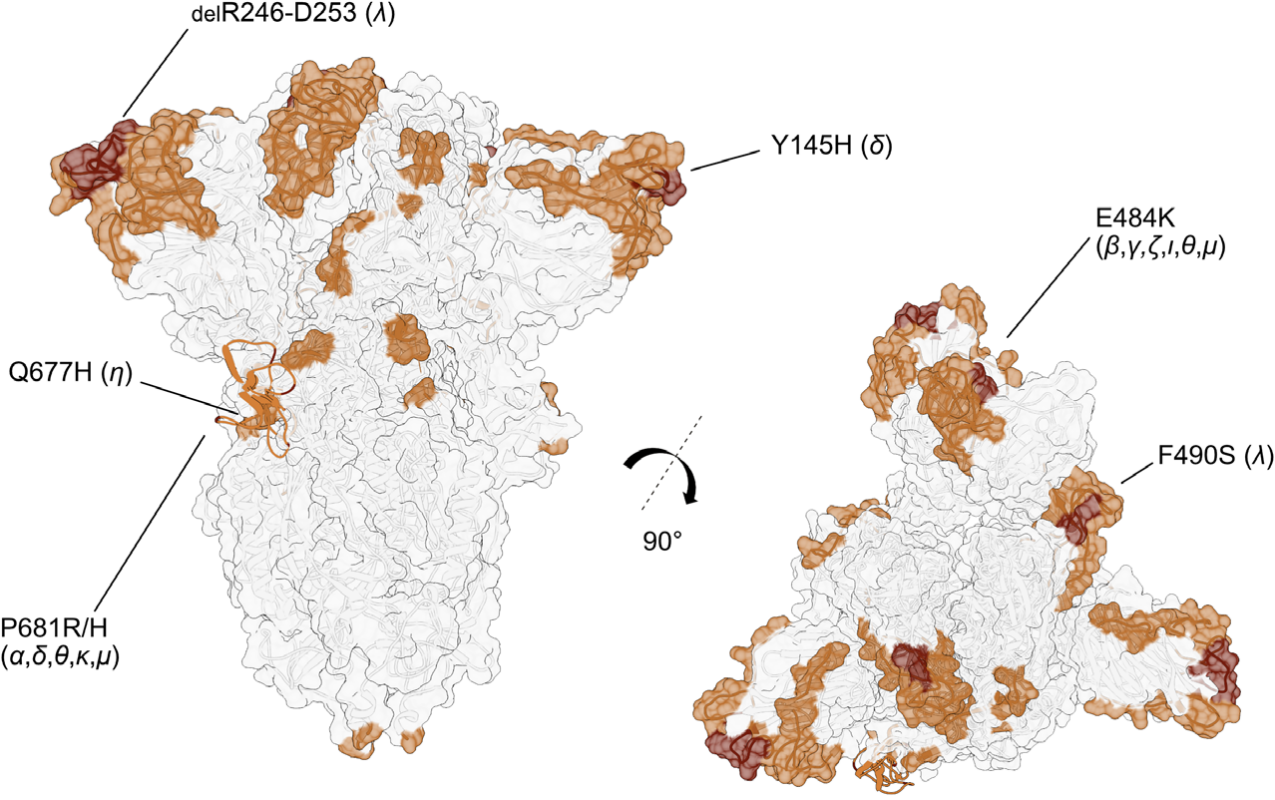
Immune escape‐related mutations mapped on the IDRs of the spike protein (structure in closed conformation) [61]. The disordered regions—according to the DisProt database (protein S, DisProt: DP02772) ‐ are coloured in light brown on the structure, while mutations are highlighted in dark brown. Molecular graphics were performed using UCSF Chimera [71].

Since late 2020, the SARS‐CoV‐2 pandemic has been driven by the emergence of variants [34]. These lineages carry fixed mutations that increase the viral fitness while enhancing the spread of the virus at population level. Our analysis reveals that nonsynonymous mutations tend to cluster in hotspots (Fig. 1,2), suggesting the presence of variable disordered regions. Such features in viral surface proteins may influence viral antigenicity and/or tropism. The external loop domain III of dengue virus envelope protein is disordered and plays a role in selective host binding ([35], DisProt: DP00876). Moreover, it is the major target of highly neutralizing and protective serotype‐specific antibodies [36]. Similarly, the HIV‐1 glycoprotein is characterized by multiple variable loops that are intrinsically disordered [37] and play a role in immune evasion [38] and coreceptor binding [39]. To assess the presence of variable disordered regions in SARS‐CoV‐2, we compared the substitutions/deletions found in the 13 major variants classified by WHO (January 2022) (https://www.who.int/en/activities/tracking‐SARS‐CoV‐2‐variants/) with the experimentally determined IDRs (Fig. 1,2,3), identifying a strong correlation among mutations and disordered regions in SARS‐CoV‐2 spike protein and nucleoprotein. For instance, mutations in the S1 subunit of the spike glycoprotein tend to cluster in hotspots at the N terminus and occur in its unstructured regions—32 out of 45 mutated positions accounting for 71% of variants are localized inside S1 IDRs, whereas the S2 chain variants do not (Table 1). Similarly, 16 out of 18 mutated positions in SARS‐CoV‐2 nucleoprotein (N) are localized inside its IDRs, accounting for 89% of variants affecting protein N (Table 1).

**Table 1:** Disorder content in SARS‐CoV‐2 proteins according to DisProt, mutation prevalence across 12 VOC and VOI lineages (except Omicron) (*mut*) and the mutations mapped to the IDRs of spike and nucleoprotein (*mut*_IDR_/*mut*). Mutations and variants data retrieved from https://outbreak.info/, intrinsic disorder data from https://disprot.org/.

	disorder content (%)	*mut*	*mut*_IDR_	*mut*_IDR_/*mut*
Spike (S1)	28	45	32	0.71
Spike (S2)	26	10	1	0.10
Nucleoprotein (N)	52	18	16	0.89

**Fig. 4:**
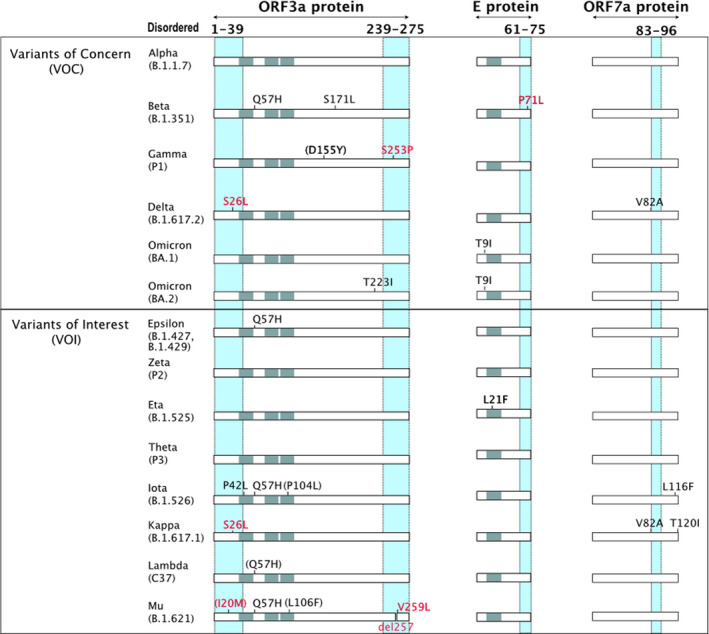
Mutations in the VOCs and VOI lineages mapped on the sequences of ORF3a, E protein and ORF7a. VOCs and VOIs lineages are represented, along with the mutations falling inside (red) and outside (black) IDRs. IDRs are represented as cyan columns while transmembrane regions are in grey.

**Fig. 5:**
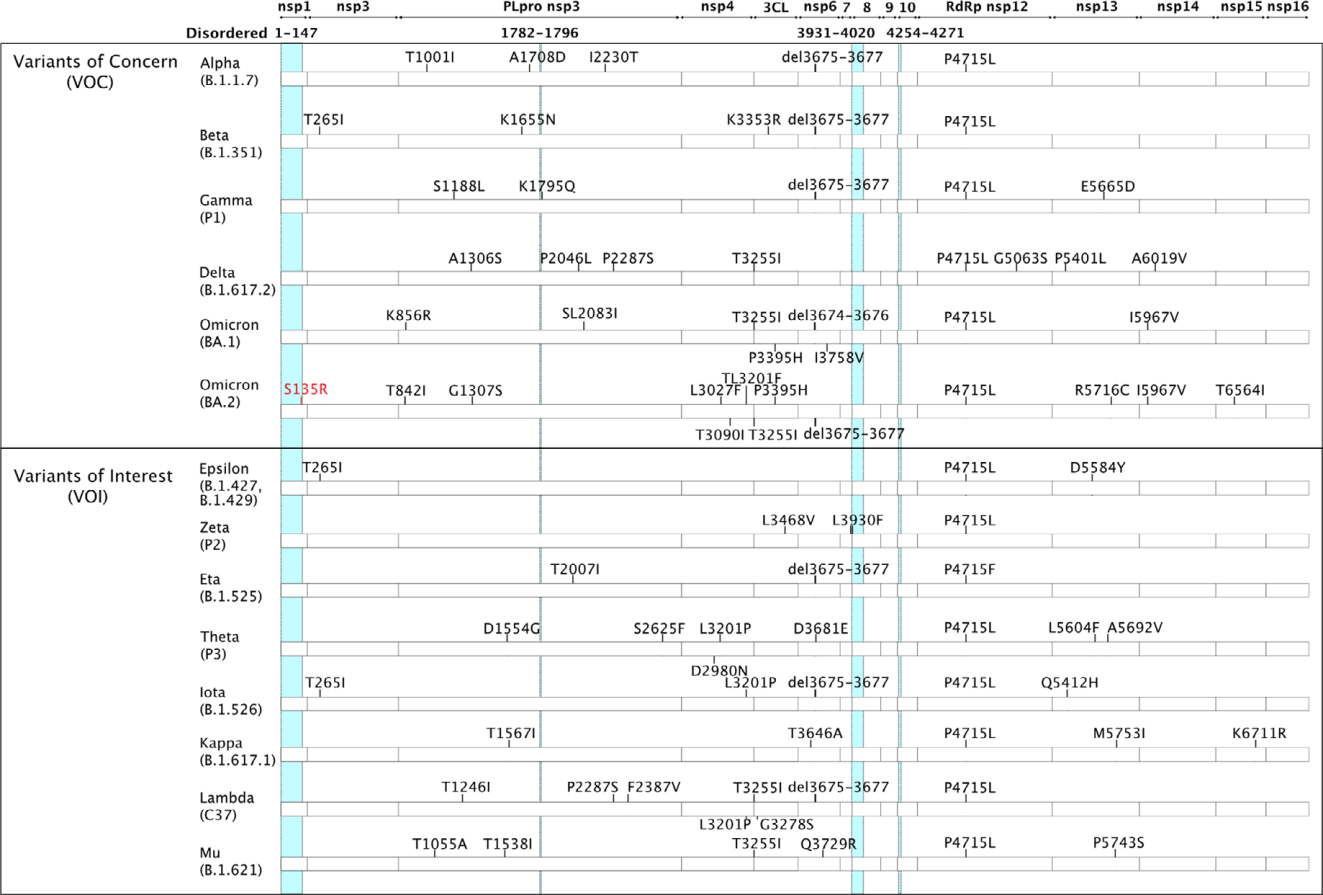
Mutations in the VOCs and VOI lineages mapped on the sequences of ORF1ab polyprotein. VOCs and VOIs lineages are represented, along with the mutations falling inside (red) and outside (black) IDRs. IDRs are represented as cyan columns.

For all the other SARS‐CoV‐2 proteins for which we gathered intrinsic disorder data, the observed mutations either did not correlate with known IDRs, or there were too few mutations to be significant. Here, we provide an insight on the intrinsic disorder and mutation content of SARS‐CoV‐2 ORF3a, E protein, ORF7a and ORF1ab (Table 2, Fig. 4,5).

**Table 2:** Disorder content in SARS‐CoV‐2 proteins according to DisProt, mutation prevalence across VOC and VOI lineages (*mut*) and the mutations mapped to the IDRs of ORF3a, E protein, ORF7a and ORF1ab (*mut*_IDR_/*mut*). Mutations and variants data retrieved from https://outbreak.info/, intrinsic disorder data from https://disprot.org/.

	disorder content (%)	*mut*	*mut*_IDR_	*mut*_IDR_/*mut*
ORF3a	28	12	5	0.42
E protein	20	3	1	0.33
ORF7a	11.6	3	0	0
ORF1ab	3.9	55	1	0.02

ORF3a (DisProt: DP03003): electron cryomicroscopy experiments of the protein shed light on the intrinsic disorder of its N‐ and C‐terminal regions [40]. Point mutations disrupting the TRAF‐binding region of ORF3a (residues 36–40) lack the ability to activate either IL‐1β or IL‐8–Luc secretion, highlighting the role of ORF3a in NF‐κB and NLRP3 inflammasome activation [41]. The ORF3a unstructured N terminus is also responsible for its subcellular localization, for instance a deletion of the first 41 residues increases its expression in the plasma membrane while impairing localization to internal membranes [40]. Finally, 42% of the mutations affecting ORF3a in the variants here described are localized in its disordered N‐ and C termini: T9I (peculiar to Omicron variant), I20M (Mu), S26L (Delta and Kappa), S253P (Gamma), del257 and V259L (Mu).

E protein (DisProt: DP03450): NMR data indicate that E, a 75‐residue‐long protein, exhibits a higher mobility in its N‐terminal (2–7) and C‐terminal (61–75) regions. The central region is characterized by structured elements, that is, a transmembrane helix (8–43) and a cytoplasmic helix (53–60) [42]. A single mutation, P71L in the Beta variant, is localized in the highly mobile C‐terminal region of the E protein.

ORF7a protein (DisProt: DP03460): X‐ray crystallography of the SARS‐CoV‐2 ORF7a ectodomain (PDB: 7CI3, residues 14–96) shows that this protein (121 aa) is characterized by a well‐defined structure and visible electron density from residues 14 to 82. Residues 83–96 are instead not visible in the electron density map, indicating the presence of structural disorder in the ORF7 protein, followed by a transmembrane domain (97–116) and an ER‐retention signal (117–121) not included in the crystal structure [43]. No mutations are found inside the IDR of ORF7a identified so far.

ORF1ab (DisProt: DP02925): Several unstructured regions were identified in the replicase polyprotein 1ab, although the structural characterization of several of its regions is still missing in the scientific literature. Residues 1–147 of ORF1ab:NSP1 are unstructured and include a flexible linker, spanning region 129–147, that connects the disordered N‐terminal domain of Nsp1 and its C‐terminal domain [44]. Similarly, IDRs are found in ORF1ab:NSP3 (residues 1782–1796), ORF1ab:NSP8 (residues 3931–4020) and ORF1ab:NSP10 (residues 4254–4271) [45, 46, 47, 48]. To date, only mutation S135R in the Omicron BA.2 lineage maps to an IDR.

### Omicron variant

During the time this paper was submitted, the Omicron variant appeared [49]. This variant is unusual in that it has more than 30 mutations localized in the spike glycoprotein, so many that it escapes most therapeutic monoclonal antibodies and, to a large extent, vaccine‐triggered antibodies [50, 51]. The variant presents a large number of mutated positions in the S1 region (*n* = 31), with a significant number mapping to disordered regions (53%) although less than the 12 previous variants (71%) (Table 3). This may be due to the tremendous acceleration of evolution that has led to omicron emergence, not yet completely understood [52]. Interestingly, in the Omicron variant and its lineages, all the mutated positions in the nucleoprotein are found in disordered regions. Specifically, P13L and del31‐33 are localized in the unstructured N terminus, while R203K and G204R are inside the intrinsically disordered linker connecting the N‐terminal domain with the C‐terminal domain. Finally, although the P13L, R203K and G204R substitutions have already been identified in other variants, the deletion affecting positions 31–33 and S413R missense mutation are peculiar to Omicron (https://outbreak.info/compare‐lineages?pango=Omicron).

**Table 3:** Disorder content in Omicron BA.1 and BA.2 SARS‐CoV‐2 proteins according to DisProt, mutation prevalence (*mut*) and the mutations mapped to the IDRs of spike and nucleoprotein (*mut*_IDR_/*mut*). Mutations and variants data retrieved from https://outbreak.info/, intrinsic disorder data from https://disprot.org/.

	disorder content (%)	*mut*	*mut*_IDR_	*mut*_IDR_/*mut*
Spike (S1)	28	39	20	0.51
Spike (S2)	26	8	0	0
Nucleoprotein (N)	52	6	6	1

### Antigenic drift is closely associated with SARS‐CoV‐2 IDRs

The major SARS‐CoV‐2‐specific antibody responses target the spike glycoprotein (S1 subunit) [8, 53]. Two major antigenic regions are present in the S1 subunit: the receptor‐binding domain (RBD) and the N‐terminal domain (NTD) [54].

The RBD is the main antigenic site to which neutralizing antibodies bind, and this region includes three IDRs. Many neutralizing antibodies target the receptor‐binding motif (RBM, pos. 438‐506) in the RBD [8, 55]. They act by preventing binding to the host receptor or reducing attachment to the host cell [54, 55]. The inner part of this region is unstructured (pos. 455–490) [7, 8] and it folds when interacting with the ACE2 receptor [22, 23].

The NTD contains an antigenic supersite to which neutralizing antibodies bind [56]. Interestingly, this supersite corresponds to the first three IDRs where most of the variation occurs [54, 57]. These three regions behave similarly to the variable loops in flavivirus envelope or HIV gp120: unstructured regions that allow the virus to escape immunity through a high potential for variation [56, 58].

Antibody recognition of disordered epitopes is particularly sensitive to epitope variation [6]. A recent study analysed viral mutations that occurred in immunocompromised patients, and found out that most mutations are observed in either the NTD supersite or the RBM [59]. The flexibility of the IDR regions allows variants to escape neutralization by many antibodies, as shown by the resistance of Beta and Gamma variants to bamlanivimab and casirivimab treatments [50]. In particular, E484K substitution—localized in the IDR within the RBM—triggers immune evasion against casirivimab monoclonal antibodies [60]. In addition, Q677H and deletion 246‐253 in the eta and lambda variants confer a better resistance to neutralizing antibodies [61].

A superantigen‐like motif—absent in other SARS family beta coronaviruses—has been identified in the spike of SARS‐CoV‐2. This motif, corresponding to the furin cleavage site at position 681–684 (PRRA) [62], was proposed to be a high‐affinity site for T‐cell receptor (TCR) β‐Chain and may play a crucial role in the immune inflammation responsible for severe cases of COVID [63]. Strikingly this motif at position 681–684 maps to an intrinsically disordered region of the spike protein, moreover P681 is a mutational hotspot in SARS‐CoV‐2 variants Alpha, Delta, Kappa, Mu (Fig. 1,3).

The nucleocapsid is the second major antigen of SARS‐CoV‐2 [64]. Early studies on SARS‐CoV showed that the immunodominant epitopes are located in regions 1–69, 153–235 and 354–422 [65], corresponding to the three disordered domains conserved in both SARS‐CoV and SARS‐CoV‐2.

Collectively, these findings suggest that the immunodominant epitopes of the S1 subunit and of the N protein are closely associated with the disordered regions in the SARS‐CoV‐2 proteins.

## Discussion

Intrinsically disordered regions (IDRs), protein regions characterized by a lack of stable three‐dimensional structure, are present and abundant in native SARS‐CoV‐2 proteins. The IDRs described here were identified by screening the associated scientific literature and the data retrieved were subsequently manually curated into DisProt and integrated with information from ViralZone. These IDRs have been shown to be associated with hotspots of mutations in spike S1 protein and nucleoprotein. Substitutions and deletions falling inside unstructured regions are likely to have a minor impact on the protein folding [66, 67]. Moreover we show that these disordered regions overlap with major antigenic sites. IDRs are known to be specific targets of antibody recognition [6] and this variability might have an impact on antibodies’ binding specificity. Our results suggest that SARS‐CoV‐2 displays disordered regions (IDRs) on the spike S1 subunit and on the N protein, and that these regions are enriched in mutations that could provide the virus with an advantage both for genetic and antigenic drift.

These findings are particularly important in light of emerging variants, such as the delta subvariant AY.4.2, which is being monitored by the European Centre for Disease Prevention and Control (ECDC, https://www.ecdc.europa.eu/) and the World Health Organization (WHO, https://www.who.int/). The major mutation associated with the AY.4.2 variant, Y145H, is located in an IDR of the spike glycoprotein and is structurally close to the known immunodominant epitope at position 153–235 (Fig. 1,3), possibly playing a role in viral immune defence. Omicron variants have a higher amount of mutations in S1 IDRs (20) than any other variants. It combines all the high‐consequence mutations identified in previous variants and has an unexpected ability to evade vaccine protection. In addition, it has an enormous number of mutations (19) in structured regions of the protein, making it distinctly different from previous variants. This suggests that Omicron arose under different selective pressures. Indeed, early studies suggest that the Omicron may have arisen in chronically infected COVID‐19 patients [52] or infected animals [68].

The proposed correlation between intrinsic disorder with mutational hotspots and major antigenic sites may have potential implications for the management of the SARS‐CoV‐2 pandemic and associated disease. Treatment of severe COVID patients depends on monoclonal antibodies, which in turn relies on their ability to recognize specific epitopes. Mutations in the targeted epitopes may inhibit the binding of monoclonal antibodies and reduce the therapeutic effect of this treatment [69]. Given the established link between IDR and mutation hotspot, it may be beneficial in the long term to select monoclonal antibodies that target ordered regions. Similarly, vaccine development could benefit from knowing where the key variable regions of the spike protein are located.

## Materials and methods

### Identification and annotation of intrinsically disordered regions

The presence of IDRs in each SARS‐CoV‐2 protein was manually curated based on the most relevant literature data as well as with manual visual inspection of crystallographic and raw structural data. In addition, we combined our annotations with information retrieved from UniProt [14], (https://www.uniprot.org/) and ViralZone [15] (https://viralzone.expasy.org/). The intrinsically disordered regions (IDRs) were then annotated in DisProt, the database for manually curated intrinsically disordered proteins [16, 17] (https://disprot.org/). Each SARS‐CoV‐2 protein described corresponds to a specific entry in DisProt: spike glycoprotein (DisProt: DP02772), nucleoprotein (DisProt: DP03212), ORF1ab (DisProt: DP02925), E protein (DisProt: DP03450), ORF7a protein (DisProt: DP03460) and ORF3a (DisProt: DP03003).

### Identification and mapping of mutations on IDRs

The analysis of SARS‐CoV‐2 mutations, both missense and deletions, relies on variants of concern (VOC), that is, Alpha, Beta, Gamma, Delta and Omicron, and variants of interest (VOI), that is, Epsilon, Zeta, Eta, Theta, Iota, Kappa, Lambda and Mu, by using the outbreak.info resource (https://outbreak.info/). Mutations with at least a minimum prevalence of 80% were considered for the analysis and then mapped on the previously identified IDRs in the spike glycoprotein and Nucleoprotein of SARS‐CoV‐2.

The trimeric spike protein structure (PDB: 6ZGG [70]) was built using Chimera to display mutations specifically affecting viral immune escape and antibody evasion [71]. Disordered region 677–689, missing from the spike structure, was modelled on the chain A starting from the sequence using RANCH [72].

## Conflict of interest

The authors declare no conflicts of interest.

## Author contributions

PLM and FQ conceived the study. ES, FQ and PLM performed the data curation and analysed the data. PLM and SCET supervised the project. FQ, ES, MC, GM, SCET and PLM contributed to writing, critically reviewing and editing the manuscript.

### Peer review

The peer review history for this article is available at https://publons.com/publon/10.1111/febs.16379.

## Data Availability

Data on intrinsically disordered regions (IDRs) can be found in DisProt (https://disprot.org/): spike glycoprotein (DisProt: DP02772), nucleoprotein (DisProt: DP03212), ORF1ab (DisProt: DP02925), E protein (DisProt: DP03450), ORF7a protein (DisProt: DP03460) and ORF3a (DisProt: DP03003). Data on SARS‐CoV‐2 variants are stored in the ViralZone resource https://viralzone.expasy.org/9556.
